# Expanding the scope of PSMA-RLT: evaluating treatment in challenging mCRPC patients with poor performance status (ECOG 3)

**DOI:** 10.1007/s00259-025-07346-4

**Published:** 2025-05-26

**Authors:** Moritz B. Bastian, Benedikt Wörl, Arne Blickle, Caroline Burgard, Tilman Speicher, Mark Bartholomä, Andrea Schaefer-Schuler, Stephan Maus, Samer Ezziddin, Florian Rosar

**Affiliations:** Department of Nuclear Medicine, Saarland University, Kirrberger Str., Geb. 50, D-66421 Homburg, Germany

**Keywords:** ECOG 3, PSMA radioligand therapy, RLT, MCRPC, Safety, Effective

## Abstract

**Purpose:**

Given the increasing inclusion of ECOG 3 patients in oncology practice, data on this subgroup in the context of prostate-specific membrane antigen (PSMA)-targeted radioligand therapy (RLT) remain limited. This study evaluates the safety and outcome of PSMA-RLT in metastatic castration-resistant prostate cancer (mCRPC) patients with ECOG performance status 3.

**Methods:**

In this analysis, a cohort of 18 mCRPC patients with ECOG performance status 3 who received PSMA-RLT was examined. The median number of treatment cycles was 2 (range: 1–10), with a mean cumulative administered activity of 21.5 ± 15.0 GBq (range: 2.7–62.6 GBq) of [^177^Lu]Lu-PSMA-617. Outcome and adverse events including hematologic and renal toxicities, fatigue, and xerostomia were analyzed.

**Results:**

50% of patients achieved either stable disease or a partial biochemical response. Median progression-free survival and overall survival were 1.3 months and 2.8 months, respectively. Severe adverse events were uncommon, occurring in three patients: one developed grade 3 leukopenia, another experienced grade 3 thrombocytopenia, and one patient had pancytopenia of grade 3/4. No higher RLT-induced grade of renal toxicity and xerostomia were observed, whilst symptoms of fatigue improved in the cohort.

**Conclusion:**

This study indicates that PSMA-RLT is a feasible and overall well-tolerated treatment for mCRPC ECOG 3 patients with manageable toxicity profile. Despite limited survival outcomes, ECOG 3 status may be considered not to be a categorical exclusion criterion for RLT. Future prospective studies should further investigate the role of PSMA-RLT in this challenging subgroup.

**Supplementary Information:**

The online version contains supplementary material available at 10.1007/s00259-025-07346-4.

## Introduction

In metastatic castration-resistant prostate cancer (mCRPC), conventional treatment strategies such as novel androgen axis drugs (NAADs), e.g. abiraterone or enzalutamide [1, 2] and taxane-based chemotherapies, including docetaxel and cabazitaxel [3–5], have been instrumental in improving survival outcomes. However, these therapies often demonstrate diminishing efficacy as the disease progresses, particularly in heavily pretreated patients with advanced disease. Radioligand therapy (RLT) targeting prostate-specific membrane antigen (PSMA) represents a further promising treatment of mCRPC. PSMA-RLT with [^177^Lu]Lu-PSMA-617 was approved by FDA and EMA based on pivotal data from the phase III VISION trial [6]. Notably, the VISION trial excluded all patients with ECOG > 2.

While the efficacy and safety of PSMA-RLT have been extensively explored in patients with good functional status [6–10], the clinical utility of this approach in individuals with poor performance status, particularly those with Eastern Cooperative Oncology Group (ECOG) performance status 3, remains underrepresented in studies and poorly understood [11–16]. ECOG 3 patients are characterized by severe functional impairment, being capable of limited self-care and confined to bed or a chair for more than 50% of waking hours [17]. This subgroup often presents unique challenges, as their reduced physiological reserves, heightened frailty, and frequent comorbidities may significantly alter treatment outcomes and toxicity profiles. The lack of dedicated investigation creates a critical gap in understanding whether [^177^Lu]Lu-PSMA-617 RLT can offer relevant therapeutic benefits to this vulnerable population or whether the risk of treatment-related toxicities outweighs potential benefits.

This study aims to systematically evaluate the efficacy and safety of PSMA-RLT in mCRPC patients with ECOG performance status 3. By analyzing this specific cohort, we aim to provide valuable insights into treatment outcomes, toxicity profiles, and the feasibility of RLT in these high-risk patients.

## Materials and methods

In this analysis, a cohort of 18 mCRPC patients with ECOG performance status 3 who received [^177^Lu]Lu-PSMA-617 PSMA-RLT was examined. Identification of patients was based on filtering for ECOG 3 within the prospective registry titled: “Prospective REgistry of Targeted RadionucLide TherapY in Patients With mCRPC (REALITY Study)” (NCT04833517). All individuals meeting this criterion were included without exclusion. Prior to initiating PSMA-RLT, all patients had undergone multiple lines of treatment, including androgen deprivation therapy (ADT), novel androgen axis drugs (NAADs), and taxane-based chemotherapy (Supplement Table S1). Comprehensive patient demographics and treatment histories are outlined in Table 1. PSMA-RLT was administered under compassionate use in accordance with § 13 (2b) of the German Pharmaceutical Act. Written informed consent was obtained from all participants following an in-depth explanation of the potential risks and side effects. Furthermore, patients provided consent for data publication in compliance with the Declaration of Helsinki. The study was approved by the local ethics committee (approval number 140/17).

**Table 1:** Patient characteristics

Patient characteristics	Value
Age	
Median [years], (range)	70 (47 – 84)
Age ≥ 65 years, n (%)	13 (72.2)
ALP, in [U/L]	
Median (range)	273 (77 – 1576)
Hemoglobin, in [g/dL]	
Median (range)	9.1 (5 – 14)
< 13.5 g/dL, n (%)	17 (94.4)
ECOG performance status, n (%)	
3	18 (100)
Prior therapies, n (%)	
Prostatectomy	6 (33.3)
Radiation	7 (38.9)
ADT	18 (100)
NAAD	18 (100)
Abiraterone	11 (61.1)
Enzalutamide	16 (88.9)
Apalutamide	4 (22.2)
Chemotherapy	13 (72.2)
Docetaxel	13 (72.2)
2nd line Cabazitaxel	5 (29.4)
[^223^Ra]Ra-dichloride	4 (22.2)
Other	2 (11.1)
PSA at baseline, in [ng/mL]	
Median (range)	305 (3.21 – 5683)
Metastases	
Bone	18 (100)
-diffuse	13 (72.2)
LN	14 (77.8)
Liver	7 (38.9)
Lung	4 (22.2)
Other (cerebral, spinal, other visceral)	4 (22.2)

*ADT* androgen deprivation therapy, *ALP* alkaline phosphatase, *ECOG* Eastern Cooperative Oncology Group, *NAAD* novel androgen axis drugs, *PSA* prostate-specific antigen, LN lymph node

### Treatment details

A total of 18 patients received PSMA-RLT, with a median of 2 cycles (range: 1–10) of [^177^Lu]Lu-PSMA-617. The activity delivered per cycle varied from 2.7 to 10.4 GBq (mean 6.8 ± 1.7 GBq), while the cumulative activity ranged between 2.7 and 62.6 GBq (mean 21.5 ± 15.0 GBq). The administered activities were adjusted individually, as previously introduced by Khreish et al. [7]. The activity administered in each cycle was chosen based on individual patient factors including tumor burden, overall disease course, extent of bone marrow involvement, general condition, and hematologic parameters. All radioligand treatments were conducted in accordance with German radiation safety regulations and carried out during inpatient hospitalization. Patients received intravenous hydration consisting of 500 mL of 0.9% sodium chloride solution and salivary gland cooling starting 30 min before the infusion. RLT was delivered intravenously through an infusion line over the course of one hour.

### Outcome analysis

Treatment response was evaluated biochemically through changes in serum PSA levels, with outcomes classified as partial remission (PR), stable disease (SD), or progressive disease (PD). PD was defined according to the Prostate Cancer Working Group 3 (PCWG3) criteria as a ≥ 25% increase in serum PSA from baseline [18]. PR was identified by a ≥ 50% reduction in serum PSA, while SD was characterized by a PSA decrease of less than 50% or an increase of less than 25%. Progression-free survival (PFS) was measured from the initiation of RLT to the occurrence of either documented progression (PSA increase > 25%), death, or the last study visit. Overall survival (OS) was defined as the time from the start of RLT until death or the most recent study visit. The follow-up period for this study was cut off on 15.09.2024.

### Adverse events

The safety of PSMA-RLT was assessed using the Common Terminology Criteria for Adverse Events (CTCAE), version 5.0 [19]. Adverse events of particular interest were anemia, thrombocytopenia, leukopenia, renal dysfunction, fatigue, and xerostomia. Blood tests were conducted regularly to monitor hematological toxicities such as anemia, thrombocytopenia, and leukopenia, as well as renal function. Fatigue and xerostomia were evaluated through structured patient interviews and questionnaires, following the criteria outlined in the CTCAE guidelines.

### Statistical analysis

Descriptive statistics were employed, and the data were found to follow a normal distribution according to the Shapiro-Wilk test. For comparisons, paired t-test was utilized. Survival outcomes were analyzed using the Kaplan-Meier method. All statistical analyses were conducted using Prism 9 software (GraphPad Software, San Diego, CA, USA) and IBM SPSS Statistics for Macintosh, version 30.0 (IBM Corp., Armonk, N.Y., USA). A *p*-value < 0.05 was considered statistically significant.

## Results

### Outcome and survival

**Fig. 1:**
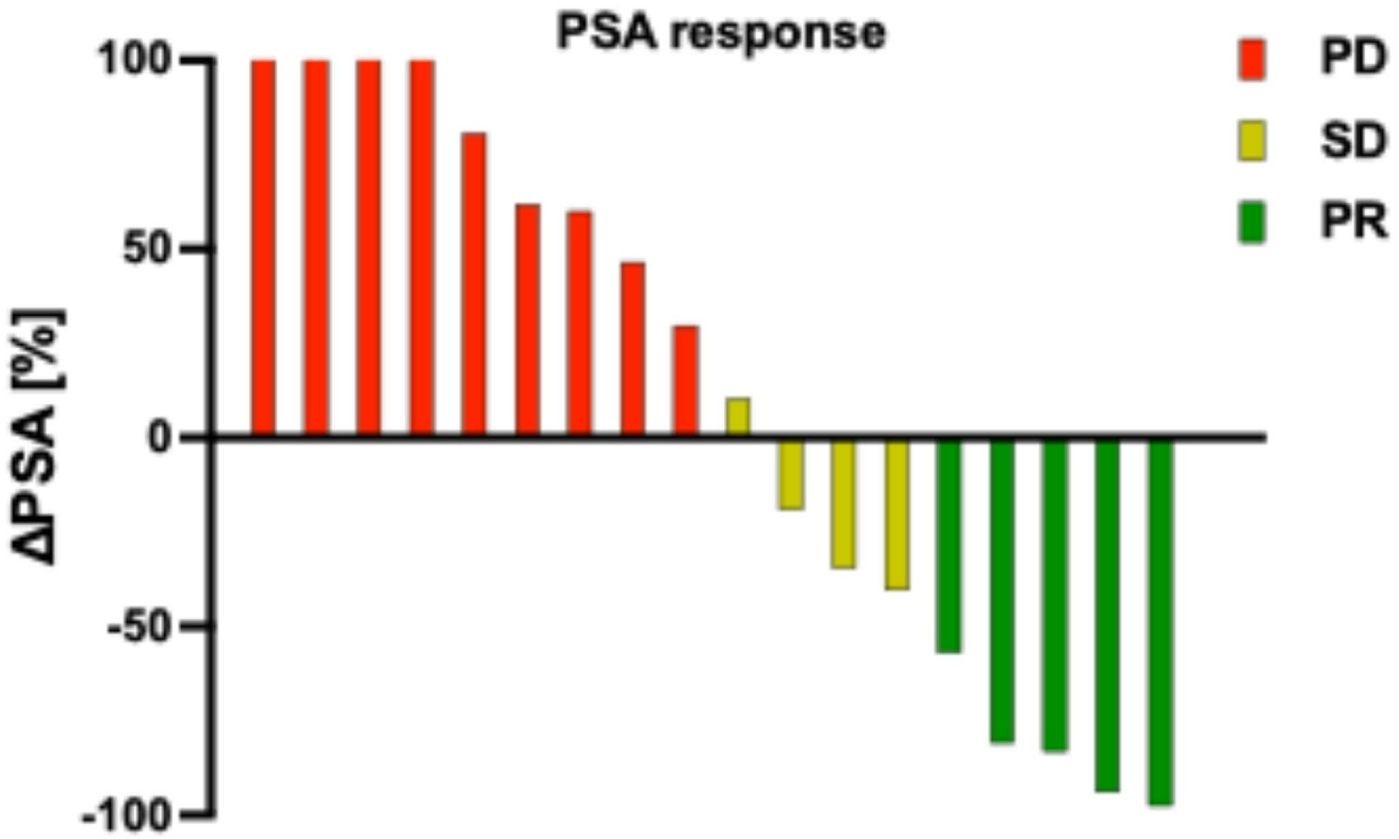
Waterfall chart showing individual change in PSA levels (ΔPSA) by treatment with PSMA-RLT, categorized into progressive disease (PD), stable disease (SD), or partial remission (PR)

**Fig. 2:**
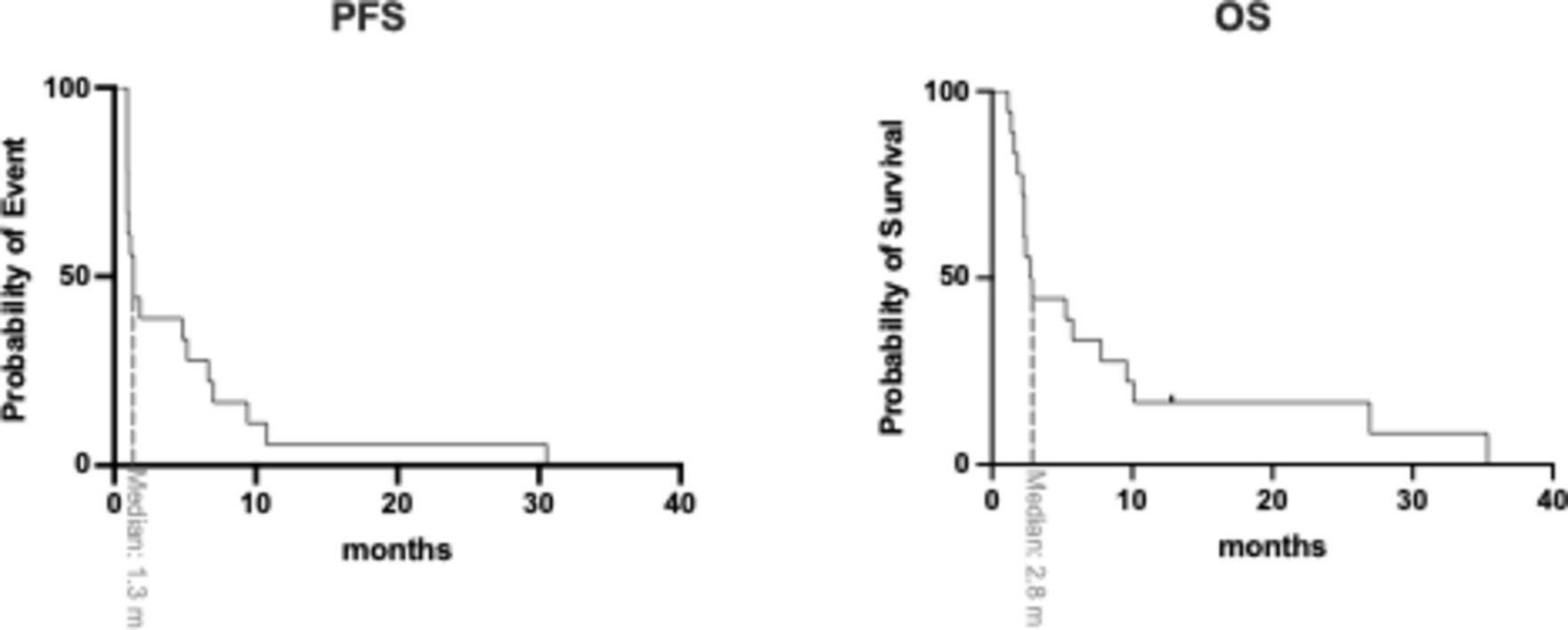
Kaplan-Meier survival plots showing PSA-based progression-free survival (PFS, left) and overall survival (OS, right) in a cohort of 18 mCRPC patients with ECOG 3 receiving PSMA-RLT

**Fig. 3:**
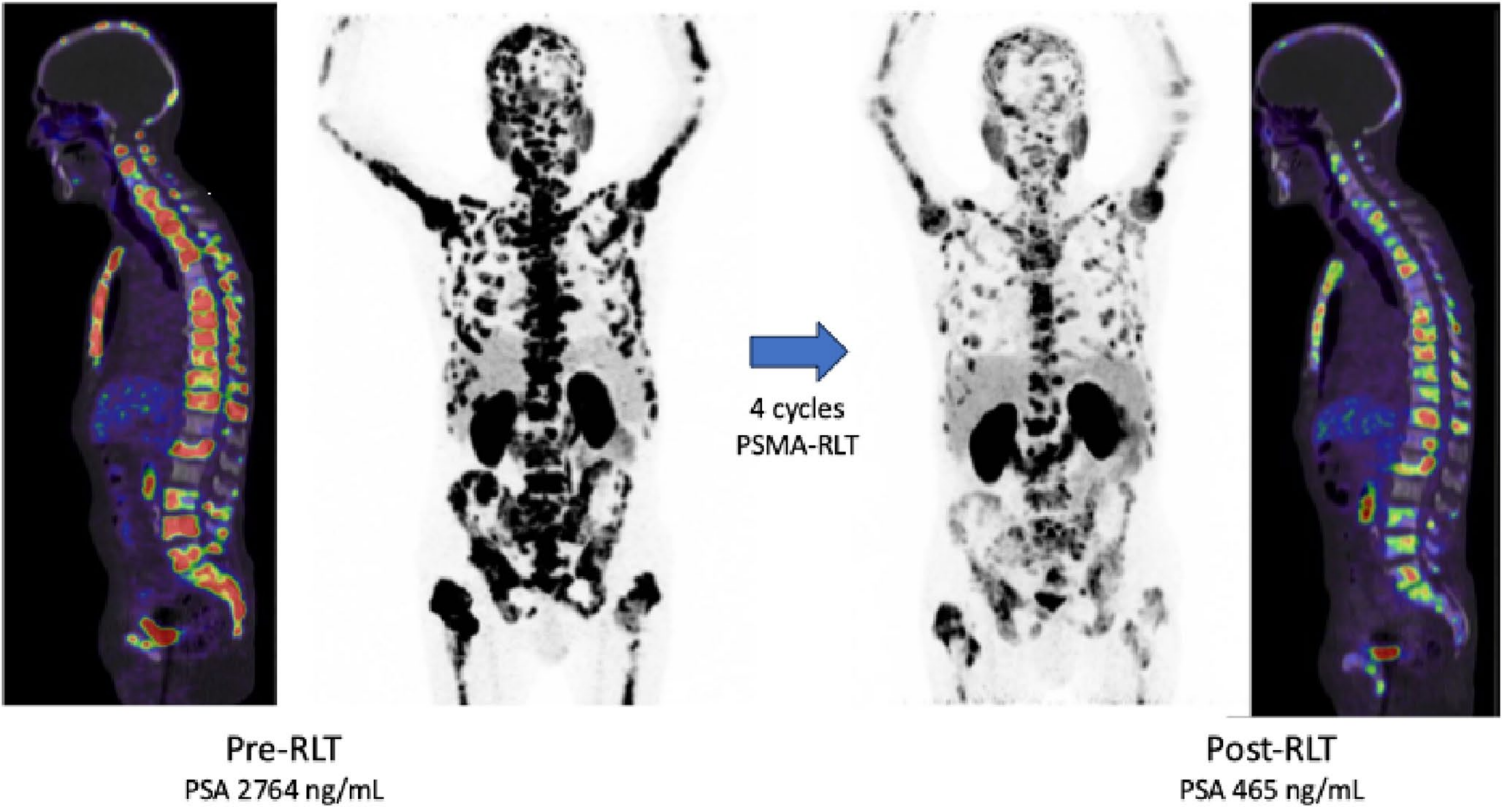
Exemplary maximum intensity projection (MIP) images showing pre-treatment and post-treatment [^68^Ga]Ga-PSMA-PET/CT scans from one patient (76 years old) treated with four cycles of [^177^Lu]Lu-PSMA-617 RLT

**Fig. 4:**
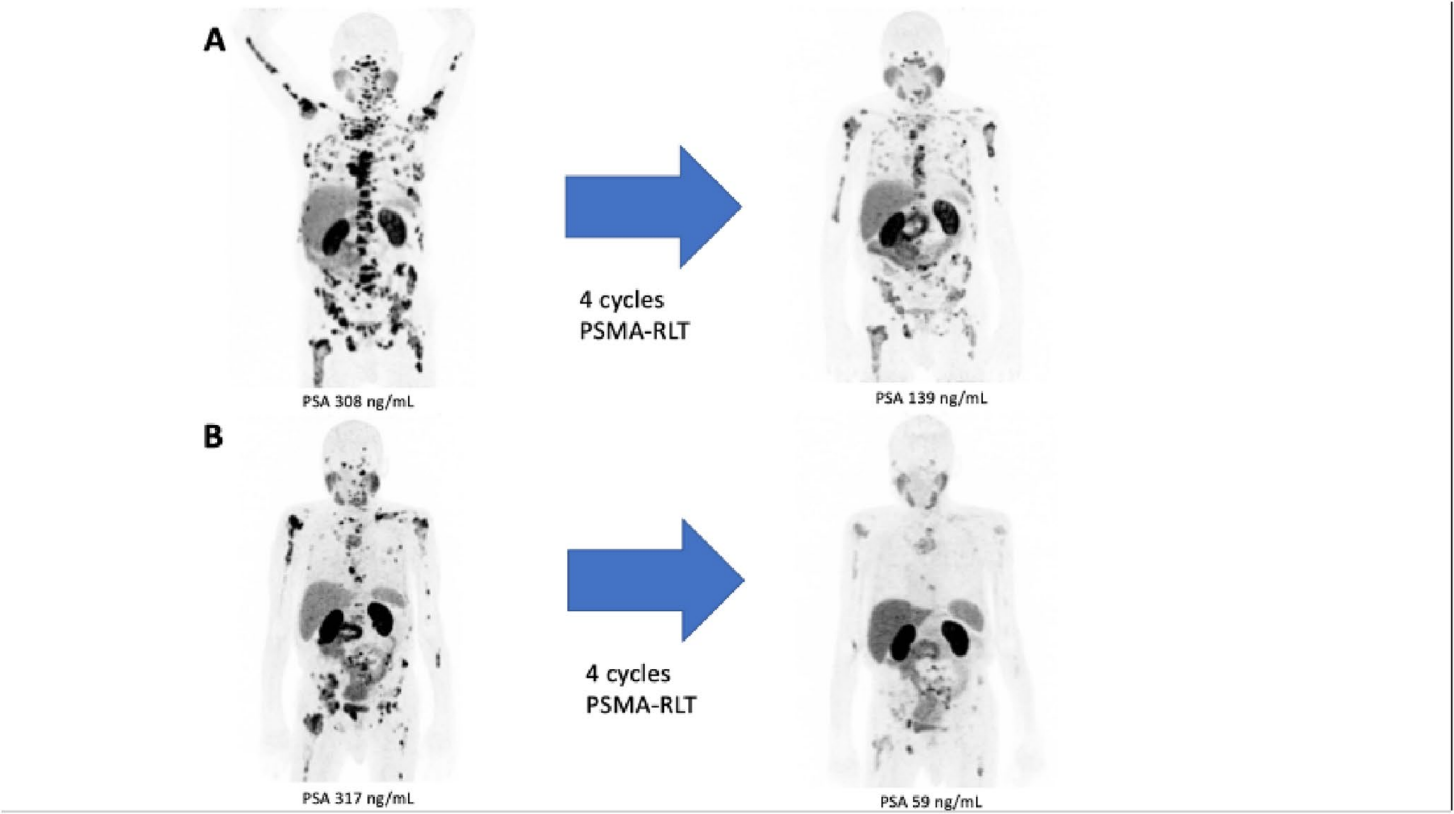
Exemplary maximum intensity projection (MIP) images showing pre-treatment and post-treatment [^68^Ga]Ga-PSMA-PET/CT scans from a patient (77 years old) after 4 cycles of [^177^Lu]Lu-PSMA-617 RLT (a, first row) and after an additional 4 cycles in a second series (b, second row), with the patient ultimately receiving a total of 10 cycles of PSMA-RLT. His PSA declined from 308 ng/mL at baseline to 139 ng/mL after 4 cycles of [^177^Lu]Lu-PSMA-617(a). However, subsequent post-RLT progression followed (PSA 317 ng/mL), prompting a second series of PSMA-RLT, achieving a nadir PSA of 59 ng/mL after 8 cycles (b); PSA subsequently increased to 93 ng/mL after the 10 th cycle

Among the 18 mCRPC patients treated with PSMA-RLT, the mean PSA change was + 33.2 ± 112.5% (median: +20.1). Of these patients, 5 (27.8%) were classified as responders, 4 (23.2%) had stable disease, and 9 (50%) showed disease progression (Fig. 1). The Kaplan-Meier analysis showed a median progression-free survival (PFS) of 1.3 months (95% CI: 0.9–1.7 months) and a median overall survival (OS) of 2.8 months (95% CI: 1.8–3.7 months). The Kaplan-Meier survival curves are shown in Fig. 2. In this cohort, 3 patients received only one cycle of PSMA-RLT, 6 patients received 2 cycles, 3 patients received 3 cycles, and the remaining 6 patients received more than 4 cycles (up to 10 cycles), with 2 patients receiving 2 series. Therapy was mostly discontinued due to disease progression or in some cases with substantial therapy response. Notably, all patients maintained their baseline ECOG performance status (3) throughout the treatment period. Illustrated in Fig. 3 is one representative patient who underwent a total of 4 cycles of [^177^Lu]Lu-PSMA-617 and in Fig. 4a patient with 2 series of PSMA-RLT.

### Adverse events

**Fig. 5:**
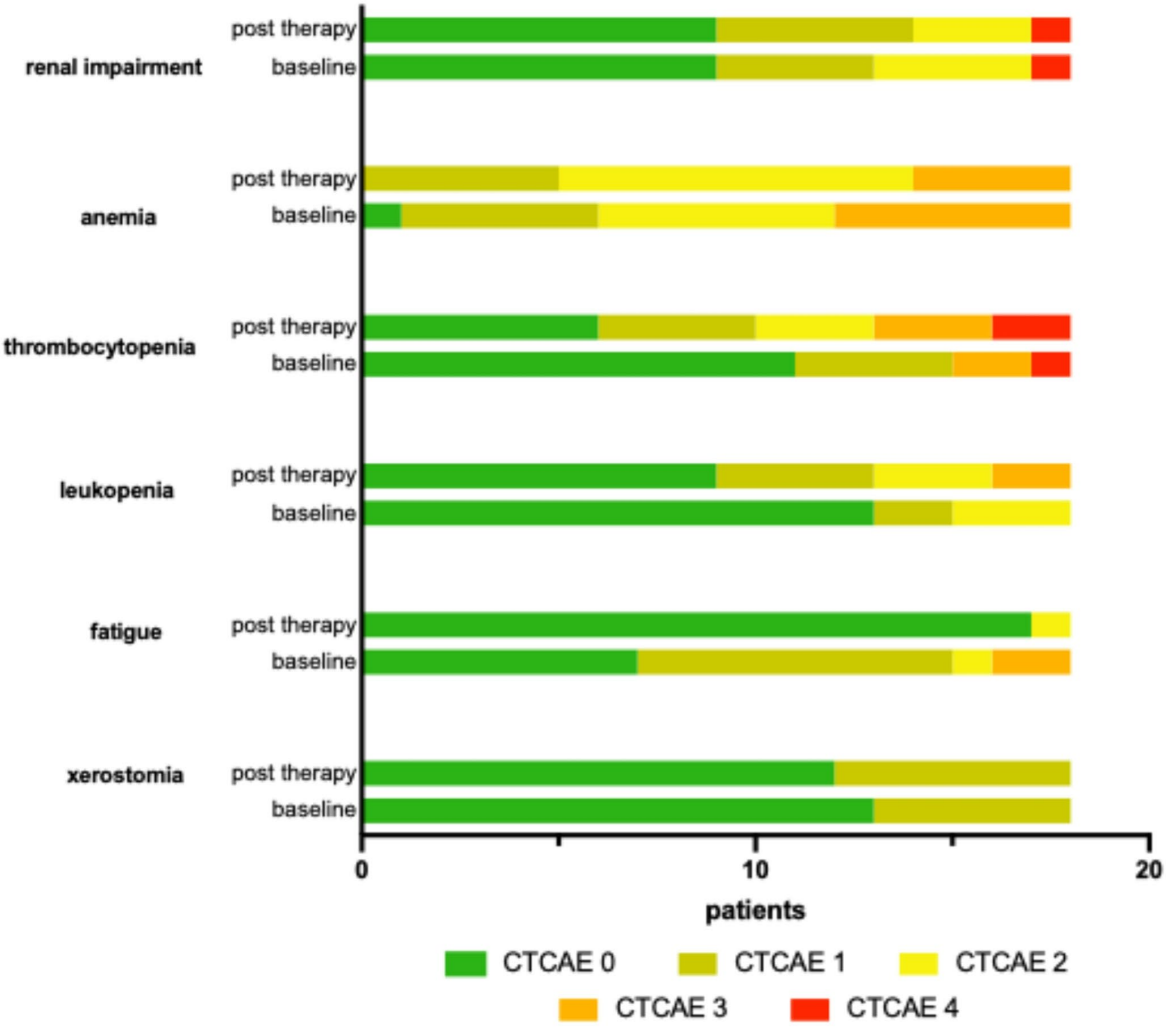
A bar graph illustrating the distribution of adverse events classified according to the ‘Common Terminology Criteria for Adverse Events’ (CTCAE), comparing occurrences before (baseline) and after PSMA-RLT

Before receiving PSMA-RLT, the average hemoglobin level was 9.32 ± 2.29 g/dL (range: 5.0–14.0 g/dL), and 8.82 ± 2.04 g/dL (range: 5.0–12.9 g/dL) after treatment (*p* = 0.243). According to CTCAE criteria, anemia was present in all 17 patients prior to therapy, with five classified as grade 1, six as grade 2 and six as grade 3. Following PSMA-RLT, anemia deteriorated in three patients, improved in four, and remained unchanged in eleven patients. After treatment, five patients had grade 1 anemia, nine had grade 2, and four had grade 3, with one newly occurred case of anemia (grade 2) (Fig. 5).

Prior to PSMA-RLT, the average leukocyte count was 6.32 ± 3.21 × 10⁹/L (range: 2.1–12.6 × 10⁹/L), and 4.64 ± 2.94 × 10⁹/L (range: 1.0–11.2 × 10⁹/L) after treatment (*p* = 0.0089). Before therapy, leukocytopenia was present in five patients, classified as CTCAE grade 1 in two cases and grade 2 in three cases. Following PSMA-RLT, the number of patients with leukocytopenia increased to nine, with six experiencing a newly occurred leukocytopenia and one patient experiencing improvement to grade 0. Post-treatment assessments showed that four patients had grade 1 leukocytopenia, three patients grade 2, and two grade 3, respectively. Notably, eight patients had normal leukocyte counts both before and after treatment (Fig. 5).

With respect to thrombocytopenia, the mean platelet count prior to PSMA-RLT was 201.7 ± 127.0 × 10⁹/L (range: 22–429 × 10⁹/L), and 108.7 ± 73.65 × 10⁹/L (range: 19–240 × 10⁹/L) following treatment (*p* < 0.0001). Seven patients exhibited thrombocytopenia before undergoing therapy, four rated as grade 1, two grade 3 and one grade 4, respectively. Following treatment, four patients presented newly occurred thrombocytopenia: three grade 1 and one grade 2. After treatment, twelve patients presented thrombocytopenia, with four classified as grade 1, three as grade 2, three as grade 3, and two as grade 4 (Fig. 5). Notably, six patients remained free of thrombocytopenia before and after PSMA-RLT.

Renal function was assessed using the glomerular filtration rate (GFR). The mean GFR was 80.15 ± 30.67 mL/min (range: 10.0–117 mL/min) before PSMA-RLT and 83.96 ± 32.12 mL/min (range: 6.3–122.0 mL/min) after treatment (*p* = 0.3521). Nine patients had renal impairment prior to therapy, with four cases classified as grade 1, four as grade 2, and one classified as grade 4. Following treatment, renal function improved in 1 patient and remained stable in 17 patients. Post-RLT, five patients exhibited grade 1 renal impairment, three had grade 2, and one showed a grade 4 impairment (Fig. 5). Notably, the patient with grade 4 renal impairment post-treatment was the same individual who had grade 4 renal impairment pre-treatment (Table S2).

Fatigue and xerostomia were assessed before and after PSMA-RLT. Fatigue was reported by 11 patients prior to treatment (eight grade 1, and one grade 2, and two grade 3) and by one patient afterwards (grade 2). Following therapy, ten patients showed improvement, one worsening, and seven remained fatigue-free throughout the entire time of the analysis (Fig. 5). Similarly, xerostomia was evaluated using CTCAE grading. Before treatment, five patients experienced xerostomia (all grade 1). After therapy six patients rated xerostomia as grade 1. 12 patients exhibited no signs of xerostomia at any point (Fig. 5).

In cases of clinically significant anemia, blood transfusions were administered as medically indicated. Platelet concentrate transfusions were not required in our cohort, and no hematopoietic growth factors were administered. One patient showed grade 3–4 pancytopenia; RLT was ultimately discontinued due to disease progression with persistent pancytopenia.

## Discussion

The findings of this study provide valuable insights into the feasibility of PSMA-RLT in a particularly challenging subgroup of mCRPC patients with poor functional status (ECOG 3). Despite extensive tumor burden and severely compromised performance status, 50% of patients achieved either a stabilization of disease or even a partial biochemical response. This highlights the potential of PSMA-RLT to achieve meaningful disease control, even in a highly challenging patient population. However, the fact that the remaining 50% showed disease progression—along with the relatively short median progression-free and overall survival—suggests that while PSMA-RLT can be administered to ECOG 3 patients, its clinical benefit in terms of PFS and OS may be limited in certain cases.

In comparison to previously published studies, to the best of our knowledge, this analysis represents the largest ECOG 3 cohort undergoing [^177^Lu]Lu-PSMA-617 RLT (> 1 cycle) reported to date. The existing literature is severely limited, typically including only single digit numbers of ECOG 3 patients per study [11–15] or only analyzing one cycle of [^177^Lu]Lu-PSMA-617 RLT in end stage mCRPC patients [16], making it difficult to draw conclusions about safety and efficacy in this fragile subpopulation. This limited evidence formed the basis for classifying ECOG 3 as a relative contraindication in the current guidelines [20]. Our findings suggest that, [^177^Lu]Lu-PSMA-617 RLT can be feasible and to some degree effective in ECOG 3 patients. However, it is important to acknowledge that the observed median PFS of 1.3 months and OS of 2.8 months in our cohort hints towards indeed worse prognosis and shorter survival outcomes than observed in patients with better functional status [6–10]. This discrepancy can certainly be attributed to the more advanced disease burden and compromised condition of our study population [21]. For instance, in the prospective VISION trial [6], Sartor et al. reported a median PFS and OS of 8.7 and 15.3 months, respectively, in a cohort containing 8.6% of ECOG 2 patients and no ECOG 3 cases. Notably, among patients with ECOG 2, survival outcomes are considerably shorter as reported in the WARMTH multicenter 617 trial [22], here patients with ECOG 2 receiving [^177^Lu]Lu-PSMA-617 only had a median OS of 6.3 months. Previous studies investigating high-risk subgroups—such as patients with extensive tumor burden and visceral metastases—have demonstrated lower response rates and shorter survival outcomes following PSMA-RLT, with reported median overall survival ranging between 6 and 11 months in these populations [23, 24]. In contrast, our cohort exhibited a markedly shorter median overall survival of only 2.8 months, highlighting the particularly advanced disease state and frailty of this patient group.

The safety profile observed underscores the feasibility of RLT in this patient cohort. Notably, nearly all patients already exhibited anemia prior to PSMA-RLT, and a substantial proportion showed pre-existing thrombocytopenia or leukopenia. This highlights the advanced disease stage and extensive tumor burden in this series, with over 70% of patients presenting diffuse bone metastases. These likely contributed to pre-treatment bone marrow compromise, which in turn possibly increased exposure to treatment-related hematologic toxicities [25]. Although a deterioration of hematologic parameters was observed in some patients following therapy, the overall rate and severity of new-onset cytopenias remained within an acceptable range, particularly considering the heavily pre-treated and functionally compromised population (one developed grade 3 leukopenia, another experienced grade 3 thrombocytopenia, and one patient had pancytopenia of grade 3/4). Furthermore, renal function remained stable in the majority of patients, indicating that nephrotoxicity did not emerge as a major concern in this vulnerable group. Overall, these findings suggest that PSMA-RLT can be safely administered to mCRPC patients with ECOG 3 performance status.

A particularly promising finding of this study is that PSMA-RLT may offer symptomatic relief for mCRPC patients with poor functional status – particularly with respect to fatigue. Fatigue is one of the most impactful symptoms in patients with reduced performance status, contributing significantly to further physical decline and impaired quality of life [26]. The observed reduction in fatigue after PSMA-RLT underscores its potential to improve both oncologic and symptomatic outcomes, enhancing functional capacity even in patients with severely compromised baseline performance. Even modest symptomatic relief may be relevant in this palliative setting, underscoring the potential importance of symptom control as a treatment goal in ECOG 3 patients receiving PSMA-RLT.

The increasing inclusion of ECOG 3 patients in oncology practice, driven by advances in supportive care and the goal of extending both life expectancy and quality of life in heavily pretreated populations, underscores the need for a deeper understanding of treatment outcomes in this vulnerable subgroup. Despite being underrepresented in clinical research, future prospective studies should aim to include ECOG 3 patients to refine therapeutic strategies. However, given the pressing need for broader patient care, clinical departments must be equipped to manage these compromised individuals. Our findings suggest that the risks of adverse events appear clearly manageable, supporting the notion that ECOG 3 status should not be an automatic exclusion criterion for PSMA-RLT. Physicians should be encouraged to consider RLT in these patients.

However, several limitations must be acknowledged. Although this cohort is larger than previously published series, the sample size remains limited, restricting generalizability. Moreover, the small sample size precluded the ability to perform statistically powerful subgroup analyses, which could have provided additional insights. Larger studies are needed to further evaluate the factors influencing adverse events in this compromised patient cohort. For the reader’s interest, we have included supplementary tables (Table S1 and S2) detailing the individual pre-treatment therapies and adverse events to allow for a more detailed association. Furthermore, this was a retrospective single-arm analysis, lacking a control group of ECOG 3 patients not receiving PSMA-RLT. Consequently, it remains uncertain whether the observed outcomes exceed those achievable with best supportive care alone. Additionally, we did not systematically assess other symptomatic outcomes (such as pain or overall quality of life), which further limits interpretation of the clinical benefit of RLT in these patients. Another limitation is that not all patients underwent [^18^F]FDG PET/CT, preventing the ability to rule out mismatch metastases which may impact outcome data. Future studies should aim to incorporate molecular imaging response assessments alongside PSA measurements.

## Conclusion

This study indicates that PSMA-RLT is a feasible and overall well-tolerated treatment modality for mCRPC ECOG 3 patients with a manageable toxicity profile. Despite limited survival outcomes, ECOG 3 status may be considered not to be a categorical exclusion criterion for RLT. As the inclusion of ECOG 3 patients in oncology practice continues to increase – driven by advancements in supportive care and the goal of improving both survival and quality of life in heavily pretreated populations – future prospective studies should specifically address this challenging and vulnerable cohort.

## Supplementary Information

Below is the link to the electronic supplementary material.

Supplementary file1 (DOCX 17.0 KB)

## Data Availability

The datasets generated during and analyzed during the current study are available from the corresponding author on reasonable request.

## Declarations

This study was in accordance with the ethical standards of the institutional research committee and with the 1964 Helsinki Declaration and its later amendments or comparable ethical standards. The study was approved by the local Institutional Review Board (ethics committee permission number 140/17).
