# Supplementary material for: Expanding the scope of PSMA-RLT: evaluating treatment in challenging mCRPC patients with poor performance status (ECOG 3)
Source: Eur J Nucl Med Mol Imaging. 2025 May 26;52(13):4893–901. doi: 10.1007/s00259-025-07346-4 (PMC12589352; doi:10.1007/s00259-025-07346-4)
Supplement: Supplementary file 1 — Supplementary file1 (DOCX 17.0 KB) [file 259_2025_7346_MOESM1_ESM.docx]

| **Patient** | **ADT** | **Enzalutamide** | **Abiraterone** | **Apalutamide** | **Docetaxel** | **Cabazitaxel** | **[^223^Ra]Ra-dichloride** |
| --- | --- | --- | --- | --- | --- | --- | --- |
| 1 | X | X | X | - | X | - | - |
| 2 | X | X | X | - | X | X | - |
| 3 | X | X | X | - | X | X | - |
| 4 | X | - | X | - | X | X | - |
| 5 | X | X | - | - | X | X | X |
| 6 | X | - | - | X | - | - | - |
| 7 | X | X | X | - | - | - | - |
| 8 | X | X | - | X | - | - | - |
| 9 | X | X | - | X | X | - | - |
| 10 | X | X | - | - | X | - | X |
| 11 | X | X | X | X | - | - | - |
| 12 | X | X | X | - | X | - | X |
| 13 | X | X | - | - | X | - | - |
| 14 | X | X | X | - | X | - | - |
| 15 | X | X | X | - | X | - | x |
| 16 | X | X | X | - | X | X | - |
| 17 | X | X | - | - | X | - | - |
| 18 | X | X | X | - | - | - | - |

Table S1: Individual treatment prior to PSMA-RLT (“X”= yes ; “-“ = No)

Table S2: Individual response according to PSA and CTCAE grading for adverse events pre and post PSMA-RLT (according to CTCAE v5.0)

| **Patient** | **Response in %** | **Cycles RLT** | **CTCAE grading pre and post therapy** | | | | | | | | | | | |
| --- | --- | --- | --- | --- | --- | --- | --- | --- | --- | --- | --- | --- | --- | --- |
|  |  |  | **Leukopenia** | | **Anemia** | | **Platelets** | | **GFR** | | **Fatigue** | | **Xerostomia** | |
|  |  |  | *pre* | *post* | *pre* | *post* | *pre* | *post* | *pre* | *post* | *pre* | *post* | *pre* | *post* |
| 1 | -40.5 | 2 | 0 | 0 | 1 | 1 | 0 | 0 | 1 | 1 | 0 | 0 | 0 | 0 |
| 2 | +180.0 | 1 | 2 | 3 | 2 | 2 | 4 | 4 | 0 | 0 | 2 | 0 | 0 | 0 |
| 3 | +123.5 | 3 | 0 | 1 | 1 | 2 | 0 | 1 | 1 | 1 | 0 | 0 | 1 | 0 |
| 4 | -57.1 | 5 | 1 | 0 | 3 | 2 | 1 | 3 | 0 | 0 | 1 | 0 | 0 | 0 |
| 5 | +10.5 | 2 | 0 | 0 | 1 | 1 | 0 | 0 | 0 | 0 | 3 | 0 | 0 | 0 |
| 6 | +266.2 | 2 | 0 | 0 | 2 | 1 | 0 | 0 | 1 | 1 | 3 | 0 | 1 | 0 |
| 7 | -83.2 | 5 | 0 | 3 | 2 | 3 | 1 | 4 | 0 | 0 | 0 | 0 | 0 | 0 |
| 8 | -97.5 | 3 | 0 | 2 | 3 | 2 | 0 | 1 | 2 | 2 | 1 | 0 | 0 | 0 |
| 9 | +46.6 | 2 | 2 | 0 | 3 | 3 | 1 | 1 | 0 | 0 | 1 | 0 | 1 | 0 |
| 10 | +61.9 | 2 | 0 | 1 | 2 | 2 | 0 | 2 | 0 | 0 | 0 | 0 | 0 | 0 |
| 11 | -94.0 | 5 | 0 | 0 | 1 | 1 | 0 | 0 | 4 | 4 | 1 | 0 | 0 | 0 |
| 12 | -19.1 | 1 | 1 | 1 | 3 | 3 | 3 | 3 | 0 | 0 | 1 | 2 | 1 | 1 |
| 13 | +80.7 | 3 | 0 | 0 | 3 | 3 | 0 | 2 | 2 | 2 | 1 | 0 | 0 | 0 |
| 14 | -34.6 | 4 | 0 | 0 | 1 | 1 | 0 | 0 | 2 | 2 | 1 | 0 | 0 | 0 |
| 15 | -80.8 | 10 | 0 | 1 | 3 | 2 | 0 | 1 | 2 | 1 | 0 | 0 | 0 | 0 |
| 16 | +29.8 | 1 | 2 | 2 | 2 | 2 | 3 | 3 | 0 | 0 | 1 | 0 | 0 | 0 |
| 17 | +60.0 | 2 | 0 | 2 | 2 | 2 | 1 | 2 | 0 | 0 | 0 | 0 | 0 | 0 |
| 18 | +247.6 | 8 | 0 | 0 | 0 | 2 | 0 | 0 | 1 | 1 | 0 | 0 | 1 | 0 |
